# Silodosin Inhibits Noradrenaline-Activated Transcription Factors Elk1 and SRF in Human Prostate Smooth Muscle

**DOI:** 10.1371/journal.pone.0050904

**Published:** 2012-11-30

**Authors:** Martin Hennenberg, Frank Strittmatter, Christer Beckmann, Beata Rutz, Claudius Füllhase, Raphaela Waidelich, Francesco Montorsi, Petter Hedlund, Karl-Erik Andersson, Christian G. Stief, Christian Gratzke

**Affiliations:** 1 Department of Urology, Ludwig-Maximilians University Munich, Munich, Germany; 2 Urological Research Institute, University of San Raffaele, Milan, Italy; 3 Wake Forest Institute for Regenerative Medicine, Wake Forest University School of Medicine, Winston-Salem, North Carolina, United States of America; University of Central Florida, United States of America

## Abstract

**Background:**

The transcription factors Elk1 and serum response factor (SRF) are central regulators of cell cycle and phenotype in various cell types. Elk1 is activated by phosphorylation (serine-383), while activation of SRF requires its co-factor, myocardin. Activation of Elk1 and SRF results in binding to specific DNA sequences in promoter regions, and may be induced by adrenergic receptor activation in different organs.

**Objective:**

To examine the effects of adrenergic stimulation on Elk1 and SRF in the human prostate and the ability of the highly selective α1A-adrenoceptor antagonist, silodosin, on transcription factor activation.

**Methods:**

Prostate tissue was obtained from patients undergoing radical prostatectomy. Expression of Elk1, SRF, and myocardin was estimated by Western blot and immunohistochemistry. Colocalizations were studied by double immunofluorescence staining. Noradrenaline- (NA-) and phenylephrine- (PE-) induced phosphorylation of Elk1 was assessed by Western blot analysis using a phospho-specific antibody. NA-induced activation of Elk1 and SRF was investigated by electrophoretic mobility shift assay (EMSA).

**Results:**

Immunoreactivity for Elk1, SRF, and myocardin was observed in stromal cells of tissues from each patient. In fluorescence stainings, SRF colocalized with myocardin and α-smooth muscle actin (αSMA). Stimulation of prostate tissues with PE (10 µM) or NA (30 µM) increased the phosphorylation of Elk1 at serine-383. NA-induced Elk1 activation was confirmed by EMSA, where a NA-induced binding of Elk1 to the DNA sequence TTTGCAAAATGCAGGAATTGTTTTCACAGT was observed. Similarly, NA caused SRF binding to the SRF-specific DNA sequence CCATATTAGGCCATATTAGG. Application of silodosin (3 µM) to prostate tissues reduced the activity of Elk1 and SRF in NA-stimulated tissues.

**Conclusions:**

Silodosin blocks the activation of the two transcription factors, Elk1 and SRF, which is induced by noradrenaline in the human prostate. A role of α1-adrenoceptors beyond smooth muscle contraction may be considered, which includes a function in transcriptional regulation.

## Introduction

Male lower urinary tract symptoms (LUTS) can be caused by benign prostate enlargement (BPE) and consequent benign prostate obstruction (BPO) [1], [2], [3]. Prostate enlargement and smooth muscle tone have for decades been regarded as separate factors contributing to LUTS in patients with BPO (“static” and “dynamic” component of obstruction) [2], [4], and medical treatment of LUTS is directed at both components. Since the dynamic component has been shown to be caused by an increased α1-adrenoceptor- (AR-) mediated prostate smooth muscle tone [5], treatment with α1-adrenoceptor antagonists is a logical first-line therapy [1], [2], [3], [5]. Recently, the novel α1-AR antagonist silodosin has been introduced in many countries [6].

However, functions of prostate α1-ARs beyond contraction have been suggested, including a role in stromal growth and prostate hyperplasia [1]. Growth and hyperplastic changes always require activation of different transcription factors. In smooth muscle cells outside the lower urinary tract, Elk1 and SRF are critically involved in cell cycle and growth [7], [8], [9], [10], [11]. Although α1-ARs, together with other factors, have been proposed to be involved in prostate growth, a regulation of Elk1 or SRF by these receptors has, to the best of our knowledge, not been considered to date.

In the present investigation, we have studied the adrenergic regulation of Elk1 and SRF transcription factors in the human prostate and its modulation by the highly selective α1A-AR antagonist, silodosin.

**Figure 1:**
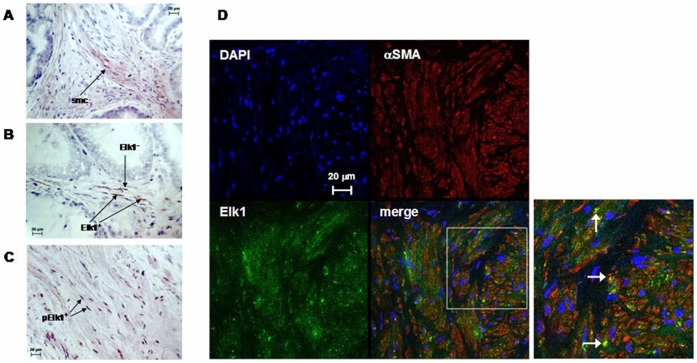
Elk1 expression in human prostate tissue. (**A**), (**B**) Peroxidase staining of prostate tissues for Elk1. (**A)** Cytosolic Elk1 immunoreactivity in smooth muscle cells (smc). (**B**) Elk1-positive (Elk1^+^) and –negative (Elk1^−^) nuclei. (**C**) Peroxidase staining of prostate tissue for phospho-Elk1, with phospho-Elk-positive (pElk1^+^) nuclei. (**D**) Double fluorescence staining of human prostate tissue for Elk1 and αSMA. Yellow color in merged pictures represents Elk1 expression in smooth muscle cells. Shown are representative pictures from stainings from tissues of n = 6 patients for each staining.

## Materials and Methods

### Human Prostate Tissue

Human prostate tissue was obtained from patients undergoing radical prostatectomy for prostate cancer, but without previous TURP (n = 60). Procedures were carried out in accordance with the Declaration of Helsinki of the World Medical Association, and has been approved by the ethics committee of the Ludwig-Maximilians University, Munich, Germany. According to the approvement of the ethics committee, informed consent from patients was not required, because all samples were collected and analyzed anonymously. All samples were taken from the periurethral zone, and did not exhibit histological signs of neoplasia, cancer, or inflammation, as assessed by a dedicated pathologist. Tissues were either directly shock frozen with liquid nitrogen, or stimulated in vitro as described below.

### In vitro Stimulation

For in vitro stimulation, tissue specimens were prepared as small strips (2–3 mm×1 mm) and allocated to dishes of a 6-well plate containing Custodiol solution. During experiments, plates were kept at 37°C under continuous shaking. For stimulation with noradrenaline (NA) or phenylephrine (PE), 10 mM stock solution was added in required intervals and volumes. Agonist concentrations used in our study (30 µM NA, 10 µM PE) are known to induce maximum contraction and activation of intracellular signaling pathways in human prostate tissue [12], [13]. To avoid effects due to different stimulation or incubation periods, samples were stimulated backwards, i. e. by addition of agonists 20 min, 10 min, and 5 min before the end of the experiment. Finally, stimulated and unstimulated samples were simultaneously shock frozen in liquid nitrogen. Therefore, all samples were exposed to the experimental conditions for identical total time periods. In separate experiments, silodosin (3 µM) or solvent (3 µl dimethylsulfoxide, DMSO) were added 15 min before application of NA (30 µM) to two of three samples. Again, all samples were exposed to the experimental conditions for identical total time periods. Samples were stored at −80°C until Western blot analysis was performed.

**Figure 2:**
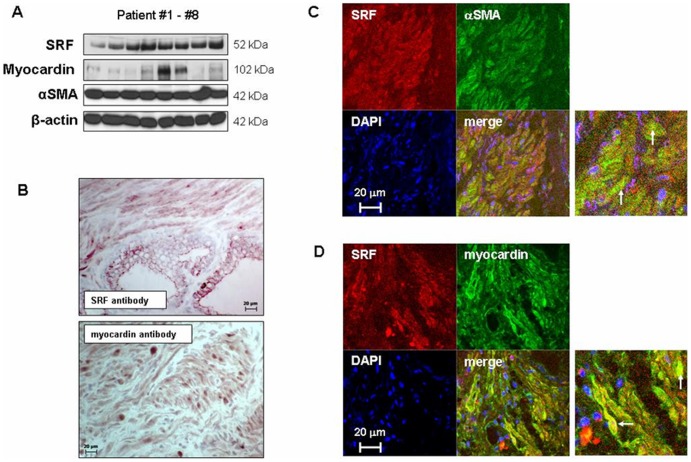
SRF and myocardin expression in human prostate tissue. (**A**) Western blot analyses with prostate tissues from n = 8 patients, showing the expression of SRF, myocardin, αSMA, and β-actin. (**B**) Peroxidase staining of prostate tissues for SRF and myocardin (representative stainings of tissues from n = 6 patients). (**C**) Double fluorescence staining of prostate tissues for SRF and αSMA (representative stainings of tissues from n = 6 patients). Yellow color in merged pictures represents SRF expression in smooth muscle cells. (**D**) Double fluorescence staining of prostate tissues for SRF and myocardin (representative stainings of tissues from n = 6 patients). Yellow color in merged pictures represents colocalization of SRF and myocardin). In (**C**) and (**D**), examples for colocalization are indicated by arrows.

**Figure 3:**
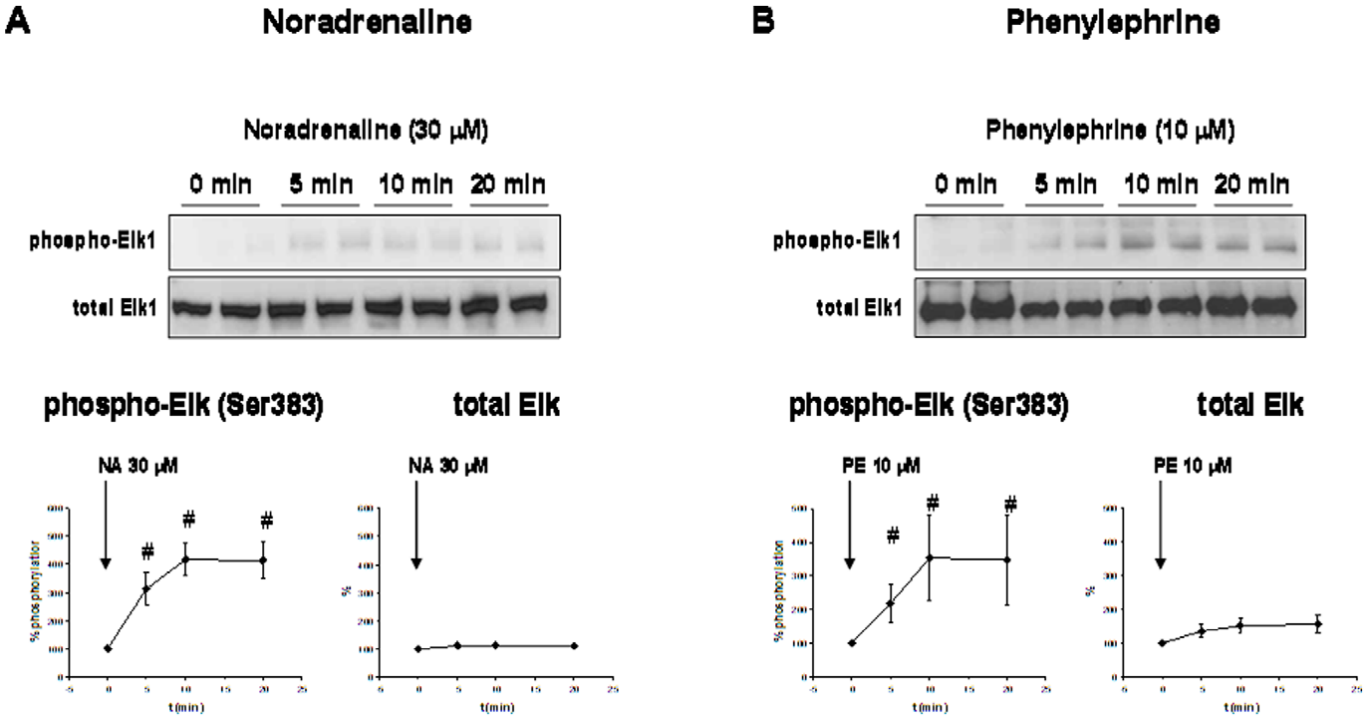
Adrenergic Elk1 phosphorylation in human prostate tissue. Prostate tissue from each patient was allocated to four samples, which were stimulated for indicated periods. Despite different stimulation periods, all samples were exposed for identical total periods to experimental conditions to prevent agonist-unspecific effects. Phosphorylation state of Elk1 and total Elk1 content was assessed by Western blot analysis. In each experiment, phospho-Elk1 and total Elk1 in unstimulated samples ( = 0 min) was set to 100%, and values for stimulated samples were referred to the unstimulated sample. (**A**) Stimulation with NA (n = 5 patients). (**B**) stimulation with the α1-AR agonist PE (n = 11 patients). Shown are representative Western blots, and densitometric quantification of all experiments (means±SEM).

### Western Blot Analysis

Frozen tissues were homogenized and analyzed by Western blot analysis as described recently [14] using the following antibodies: monoclonal mouse anti phospho-Elk1 (serine-383) (B-4), monoclonal mouse anti Elk1 (3H6D12), monoclonal mouse anti SRF (A-11), polyclonal rabbit anti myocardin, monoclonal mouse anti α-smooth muscle actin (αSMA), or monoclonal mouse anti β-actin (C4) (all from Santa Cruz Biotechnology, Santa Cruz, CA, USA). Blots were developed with enhanced chemiluminescence (ECL) using ECL Hyperfilm (GE Healthcare, Freiburg, Germany). Intensities of resulting bands were quantified using Image J (NIH, Bethesda, Maryland, USA). In experiments for expression analysis, tissues from eight patients were assessed in each gel. In stimulation experiments, samples without and with agonist were semiquantitatively compared: samples without stimulation were set to 100%, and stimulated samples from the same prostate were expressed as % of unstimulated samples.

### Immunohistochemistry

Sections (6–8 µm) from frozen tissues were stained by an indirect immunoperoxidase technique, as described recently [14] using the following antibodies: monoclonal mouse anti phospho-Elk1 (serine-383) (B-4), monoclonal mouse anti Elk1 (3H6D12), monoclonal mouse anti SRF (A-11), or polyclonal rabbit anti myocardin (all from Santa Cruz Biotechnology, Santa Cruz, CA, USA). Control stainings without primary antibodies did not yield any immunoreactivity.

### Immunofluorescence

Human prostate specimens were double labelled as described recently [14] using the following antibodies: monoclonal mouse anti Elk1 (3H6D12), monoclonal mouse anti SRF (A-11), polyclonal rabbit anti myocardin (all from Santa Cruz Biotechnology, Santa Cruz, CA, USA), and rabbit polyclonal anti α-smooth muscle actin (αSMA) (Thermo Scientific, Waltham, MA, USA). Binding sites were visualized using Cy3 and Cy5 conjugated secondary antibodies (goat anti mouse, AP124C, Millipore, Billerica, MA, USA; goat anti rabbit, ab6564, Abcam, Cambridge, UK). Nuclei were counterstained with DAPI (Invitrogen, Camarillo, CA, USA). Fluorescence was recorded with separate detectors. Control stainings without primary antibodies did not yield any signals.

### EMSA

Activation of Elk1 and SRF was investigated by non-radioactive electrophoretic mobility shift assays (EMSA), where binding of transcription factors to biotin-labelled, specific DNA probes is determined. Assays were performed using commercially available kits (Affymetrix, Santa Clara, CA, USA) according to the manufacturer’s instruction. In brief, stimulated and unstimulated tissues were homogenized and subjected to protein determination. Twenty µg of protein were incubated with biotin-labelled DNA probe with the sequence TTTGCAAAATGCAGGAATTGTTTTCACAGT (5′3′) for Elk1, or CCATATTAGGCCATATTAGG for SRF. After incubation, samples were subjected to electrophoresis in native, non-denaturating acrylamide gels (6%), and subsequently blotted on nylon membranes, where detection for biotin was performed with peroxidase-coupled streptavidin and ECL. Intensities of resulting bands were quantified using Image J (NIH, Bethesda, Maryland, USA). Correct experimental conditions were approved by application of a negative control provided by the manufacturer.

**Figure 4:**
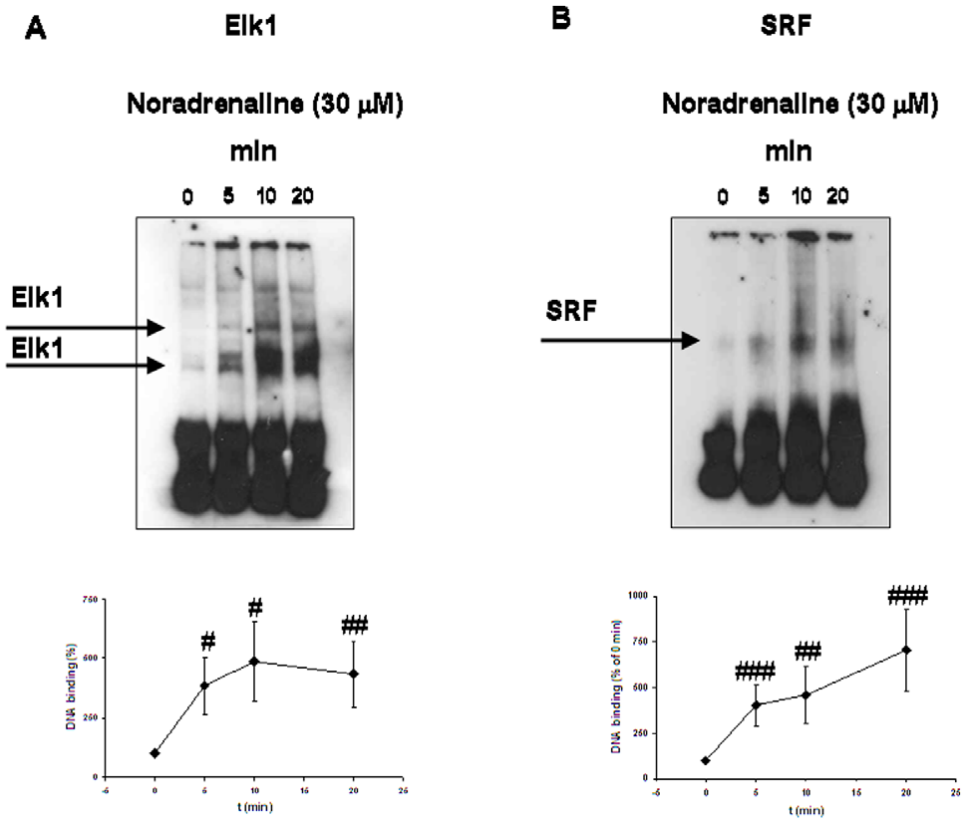
NA-induced Elk1 and SRF activation in human prostate tissue. Prostate tissue from each patient was allocated to four samples, which were stimulated for indicated periods. Despite different stimulation periods, all samples were exposed for identical total periods to experimental conditions to prevent agonist-unspecific effects. Elk1 and SRF activities were asssessed by EMSA. Bands for active transcription factors (bound to DNA probes) were identified using negative controls, and are indicated by arrows. In each experiment, Elk1 or SRF in unstimulated samples ( = 0 min) was set to 100%, and values for stimulated samples were referred to the unstimulated sample. (**A**) NA-induced Elk1 activation (n = 9 patients). (**B**) NA-induced SRF activation (n = 9 patients). Shown are representative experiments, and densitometric quantification of all experiments (means±SEM).

**Figure 5:**
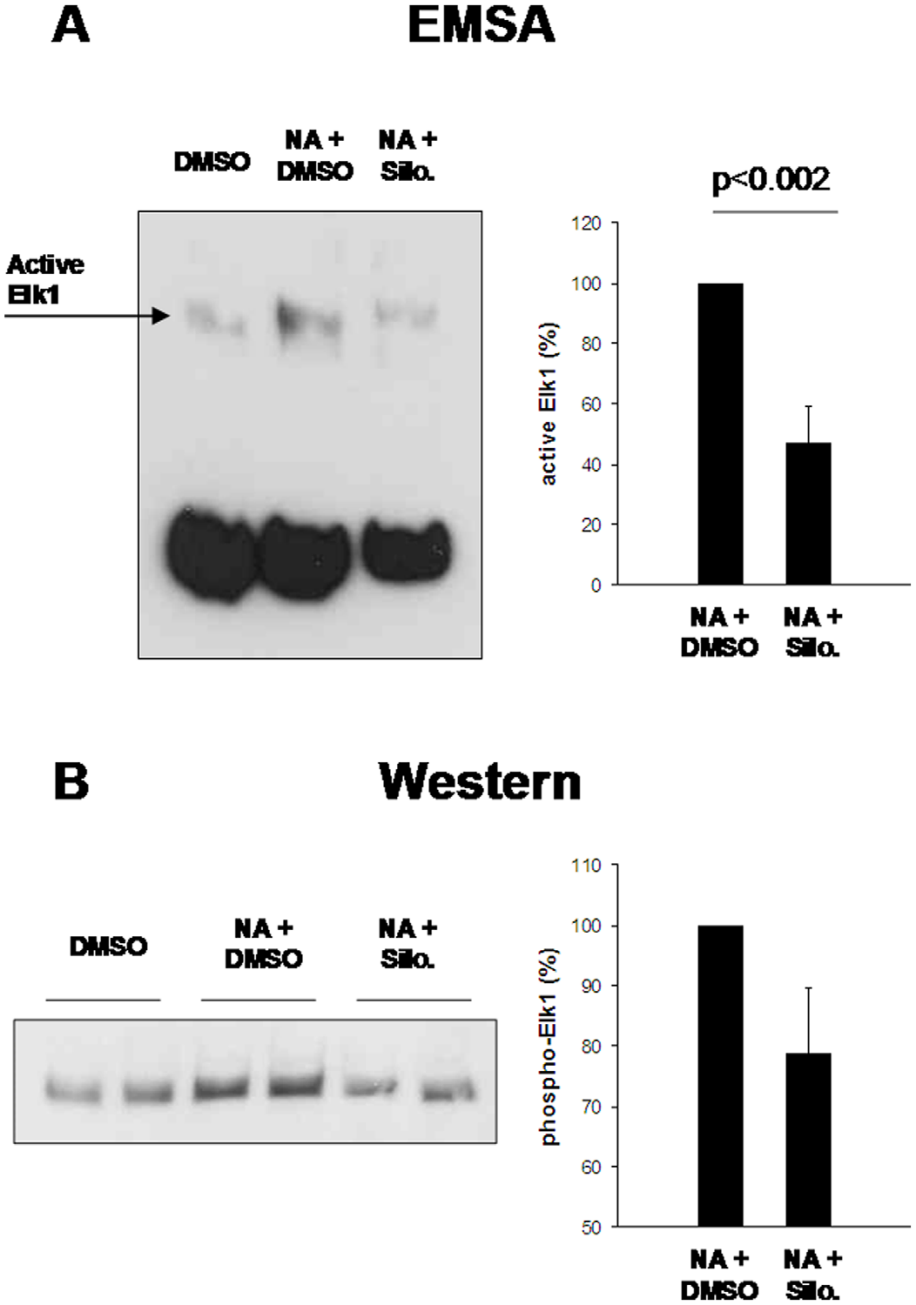
Inhibition of NA-induced Elk1 activation by silodosin. Prostate tissue from each patient was allocated to three samples, which were stimulated with NA (30 µM) for 15 min, or remained unstimulated. Silodosin (3 µM) or solvent (3 µl DMSO) were added 15 min before NA as indicated. All samples were exposed for identical total periods to experimental conditions to prevent agonist-unspecific effects. In each experiment, Elk1 in NA-stimulated samples without silodosin ( =  DMSO) was set to 100%, and values for samples with silodosin were referred to that sample. (**A**) Inhibition of Elk1 activity by silodosin in NA-stimulated prostate samples (n = 6 patients), detected by EMSA. (**B**) Inhibition of Elk1 phosphorylation by silodosin in NA-stimulated prostate samples (n = 6 patients), assessed by Western blot analysis. Shown are representative experiments, and densitometric quantification of all experiments (means±SEM).

**Figure 6:**
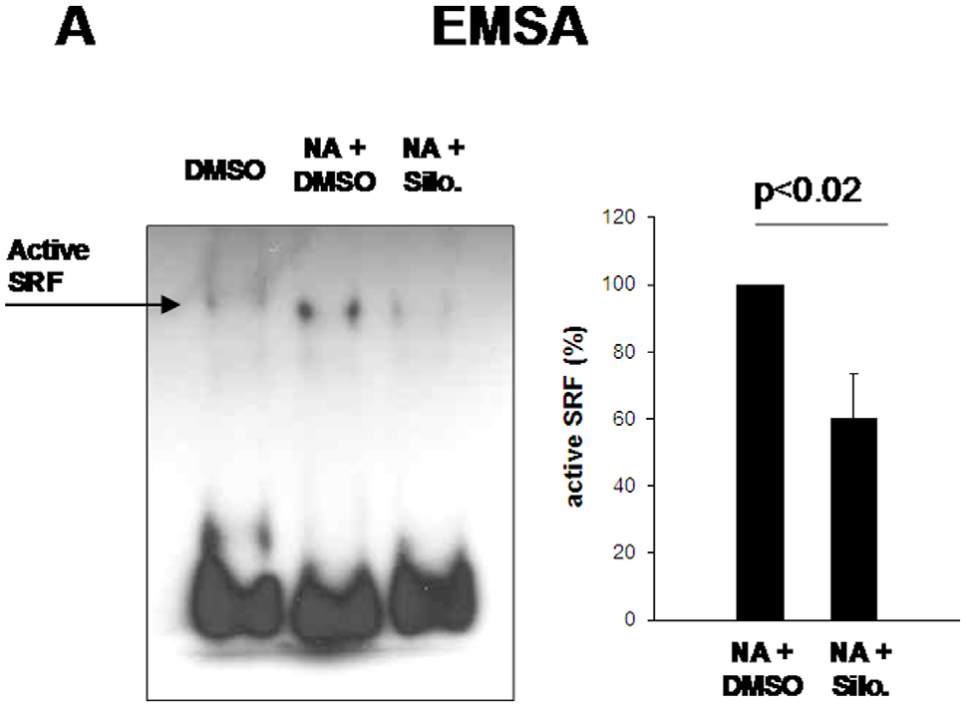
Inhibition of NA-induced SRF activation by silodosin. Prostate tissue from each patient was allocated to three samples, which were stimulated with NA (30 µM) for 15 min, or remained unstimulated. Silodosin (3 µM) or solvent (3 µl DMSO) were added 15 min before NA as indicated. All samples were exposed for identical total periods to experimental conditions to prevent agonist-unspecific effects. In each experiment, SRF in NA-stimulated samples without silodosin ( = DMSO) was set to 100%, and values for samples with silodosin were referred to that sample. Shown are representative experiments, and densitometric quantification of all experiments (n = 6 patients; means±SEM).

### Drugs and Solutions

Aqueous stock solutions for NA and the α1-AR agonist PE (Sigma, St. Louis, MO, USA) (10 mM) were freshly prepared before each experiment. Silodosin, a highly selective α1A-AR antagonist [6], [15] was kindly provided by Recordati S. p. A. (Milan, Italy). Silodosin was added as 10 mM stock solution in DMSO, which was stored at −20°C.

### Statistical Analysis

Data are presented as means±standard error of the mean (SEM) with the indicated number (n) of experiments. Two-tailed student *t* test was used for paired or unpaired observations. *P* values <0.05 were considered statistically significant.

## Results

### Elk1 Expression

After peroxidase staining with an Elk1 antibody, immunoreactivity was observed in samples from each investigated patient (n = 6). Imunoreactivity was observed in stromal cells, but not in epithelial cells (Fig. 1A). Elk1 immunoreactivity was located to the cytosol and nuclei (Fig. 1A,B). Similarly, peroxidase staining with a phospho-specific Elk1 resulted in immunoreactivity in each investigated prostate sample (n = 6 patients). Immunoreactivity for phospho-Elk1 was observed in stromal cells, where it was located to the cytosol and nuclei (Fig. 1C).

Fluorescence staining of prostate samples (n = 6 patients) with antibodies for Elk1 or αSMA resulted in immunoreactivity in the prostate stroma (Fig. 1D). In merged pictures, Elk1 and αSMA showed discrete colocalization, as indicated by yellow color in the prostate stroma after overlay (Fig. 1D).

### SRF and Myocardin Expression

Western blot analysis for SRF revealed bands matching the expected size (52 kDa), which were observed in prostate samples from each investigated patient (n = 8) (Fig. 2A). Peroxidase staining of prostate samples (n = 6 patients) using a SRF antibody resulted in immunoreactivity in stromal cells, which was observed in each investigated sample (Fig. 2B). Similarly, Western blot analysis for myocardin revealed bands matching the expected size (102 kDa) in prostate samples from each investigated patient (n = 8) (Fig. 2A). Peroxidase staining of prostate samples (n = 6 patients) using a myocardin antibody resulted in immunoreactivity in stromal cells, which was observed in each investigated sample (Fig. 2B). The smooth muscle marker, αSMA, and the housekeeping protein and loading control, β-actin, was detectable by Western blot analysis in samples of each investigated patient (n = 8) (Fig. 2A). The content of αSMA and β-actin was similar between these samples (Fig. 2A).

Double fluorescence staining of prostate samples (n = 6 patients) with antibodies for SRF and αSMA resulted in immunoreactivity in the prostate stroma (Fig. 2C). In merged pictures, SRF and αSMA showed colocalization, as indicated by yellow color in the prostate stroma after overlay (Fig. 2C). Similarly, fluorescence staining of prostate samples (n = 6 patients) with a myocardin antibody resulted in immunoreactivity in the prostate stroma (Fig. 2D). Fluorescence for myocardin colocalized with immunoreactivity for SRF, as indicated by yellow color in merged pictures (Fig. 2D).

### Elk1 Phosphorylation by Adrenergic Stimulation

By Western blot analysis, phospho-Elk1 and total Elk1 were detected with the expected molecular weight (45 kDa) in prostate samples. Stimulation of prostate samples (n = 5 patients) with NA (30 µM) caused Elk1 phosphorylation at serine-383, as indicated by increased density of bands for phospho-Elk1 (Fig. 3A). NA-induced Elk1 phosphorylation was observed 5, 10, and 20 min after stimulation (Fig. 3A). The content of total Elk1 in these samples remained unchanged, as shown by Western blot analyses using a non-phospho-specific Elk1 antibody (Fig. 3A). Similarly, stimulation of prostate samples (n = 11 patients) with PE (10 µM) caused Elk1 phosphorylation at serine-383, which was observed 5, 10, and 20 min after stimulation (Fig. 3B). The content of total Elk1 in these samples remained unchanged (Fig. 3B).

### Elk1 and SRF Activation by NA

Stimulation of prostate tissues (n = 9 patients) with NA (30 µM) increased the binding of Elk1 to the Elk1-specific DNA probe with the sequence TTTGCAAAATGCAGGAATTGTTTTCACAGT, indicating NA-induced Elk1 activation (Fig. 4A). Similarly, stimulation of prostate tissues (n = 9 patients) with NA (30 µM) induced SRF activation, as shown by increased binding of SRF to the SRF-specific DNA probe with the sequence CCATATTAGGCCATATTAGG after NA stimulation (Fig. 4B). NA-induced activation of Elk1 and SRF was observed after 5, 10, and 20 min of stimulation (Fig. 4).

### Inhibition of NA-induced Elk1 and SRF Activation by Silodosin

Application of silodosin (3 µM) to prostate tissues (n = 6 patients) 15 min before addition of NA (30 µM, 15 min) reduced the content of activated Elk1 in EMSA (Fig. 5A), and of phosphorylated Elk1 in Western blot analysis (Fig. 5B). Similarly, silodosin reduced the content of SRF in NA-stimulated samples (n = 6 patients), as detected by EMSA (Fig. 6).

## Discussion

The present results show that in the human prostate, α1-AR stimulation with NA activates the two transcription factors, Elk1 and SRF, and that the selective α1A-AR antagonist, silodosin, blocks this activation. To the best of our knowledge, this is the first evidence that stimulation of α1-ARs causes activation of transcription factors in human prostate smooth muscle.

Activation of transcription factors is mandatory for growth- and differentiation-related processes, which are the basis for hyperplastic changes. Therefore, we assume that α1-AR-mediated Elk1 and SRF activation may be involved in prostate growth and hyperplasia. Contraction and growth may both contribute to BPO, but were regarded as separate phenomenons for decades (“dynamic” and “static component”) [2], [4]. α1-AR activation of Elk1 and SRF may thus represent a connection linking adrenergic contraction to growth/hyperplasia. Whether both factors act in concert or separately in the prostate, may not be concluded from our present findings. Detailed identification of Elk1 and SRF function in the prostate require studies in genetically modified cell cultures or animals. Specific pharmacological inhibitors for Elk1 or SRF have not been developed to date. Therefore, this remains a large issue, which may be adressed by separate studies.

Our study was performed in tissues from patients undergoing radical prostatectomy. We used non-malignant tissue from the periurethral zone, while most prostate tumors are located to the peripheral zone [16], [17]. Almost all patients undergoing radical prostatectomy show prostate hyperplasia. Therefore, normal, non-hyperplastic tissue was not available for our study, and our findings may represent the conditions in the hyperplastic prostate. Of note, expression of the smooth muscle marker αSMA was detectable in all investigated samples.

Elk1 is activated by phosphorylation at serine-383, resulting in Elk1 binding to specific DNA sequences of promoter regions [10], [18]. These sequences are mimicked by biotin-labelled DNA probes in EMSA, while the phosphorylation can be investigated using phospho-specific antibodies. Using both techniques, we observed Elk1 activation by PE and NA that could be counteracted by silodosin. Transcriptional regulation by transcription factors requires their nuclear localization, so that Elk1 is recruited from the cytosol to nuclei after its activation [10], [18]. In our immunohistochemical stainings, many smooth muscle cells showed Elk1- and phospho-Elk1-positive nuclei. This supports the idea that parts of the prostatic Elk1 population are active, and regulate transcription under physiological conditions. However, we can not exclude the contribution of non-adrenergic mediators to prostate Elk1 activity besides α1-ARs. In cultured prostate cancer cells, androgen receptors and growth factors activate Elk1 as well [19], [20], [21].

In smooth muscle cells outside the lower urinary tract, Elk1-dependent transcription has been related to proliferation, growth and differentiation [8], [9], [10]. Elk1 was identified as an effector of the extracellular signal regulated kinases (ERK1/2), which are ubiquitous regulators of cell cycle [8], [9], [10], [18]. Interestingly, stimulation of α1-ARs in human prostate tissues or cultured prostate smooth muscle cells results in activation of ERK1/2 [12], [22]. Similar to non-malignant conditions, Elk1 activation in prostate cancer cells is involved in proliferation and tumor growth [20], [23].

Using a SRF-specific EMSA, we observed that stimulation of prostate α1-ARs activates SRF in addition to Elk1. Activation of SRF requires the cofactor myocardin [7], [11]. Expression of myocardin and its colocalization with SRF in our prostate samples was confirmed by double fluorescence stainings. In smooth muscle cells of the cardiovascular system, SRF connects adrenergic contraction with cellular differentiation and proliferation [7], [11]. This link has been termed as “transcription-excitation coupling” [7], [11]. By activation of SRF, adrenergic receptors are involved in the regulation of smooth muscle cell phenotype [7], [11]. Cells may switch between a “contractile” or “synthetic” phenotype; each phenotype being characterized by different contractility and proliferation rate [7], [11]. Our present findings suggest that a similar, SRF-mediated coupling of contraction to cellular differentiation may exist in human prostate smooth muscle.

It is widely accepted that α1-AR antagonists cause improvement of symptoms by smooth muscle relaxation in the lower urinary tract, including the prostate [1], [2], [3], [5]. In addition to its role for contraction, non-motoric functions mediated by prostate α1-ARs have been previously assumed by different authors [1], [4]. Studies performed in animal models and cultured cells, but also clinical data suggested an involvement of prostate α1-adrenoceptors in prostate growth and hyperplasia [24], [25]. Intervention of α1-blockers into the cell cycle was observed in cultured prostate cells, but also in patients receiving α1-AR antagonists [26], [27], [28], [29], [30], [31]. The latter was associated with reduced growth and stromal regression [27], [28], [29], [30], [31]. However, substantial regression of prostate volume has not been demonstrated during the widespread application of α1-AR antagonists. Consequently, it has been proposed that α1-ARs act in concert with hormones and growth factors, being just one of the factors within a complex regulatory network that regulates prostate growth [12]. Nevertheless, a reduction of prostate volume was observed in a clinical study using terazosin [32]. In contrast, therapy with alfuzosin for three month did not reduce prostate volume [33], nor did the long-term treatment in the MTOPS (doxazosin) or COMBAT (tamsulosin) study [34], [35]. It has been proposed that further studies are required, as effects may be substance-specific or depend on study conditions [33].

The effect of silodosin on prostate volume has not been considered to date. Since the majority of the stromal α1-ARs are of the α1A subtype [1], particularly in BPH, the present results were not unexpected. Considering its high selectivity for α1A-ARs [15], it cannot be excluded that the effects of silodosin may differ from that of other α1-ARs antagonists. Therefore, further investigations are required.

### Conclusions

α1-AR-mediated activation of Elk1 and SRF may represent a mechanism connecting prostate contraction and growth (“dynamic” and “static” components) to each other. The function of prostate α1-ARs is not confined to smooth muscle contraction, but also comprises regulation of transcriptional activity. Adrenergic Elk1 and SRF activation in the human prostate can be blocked by silodosin. The translational value of this silodosin effect needs to be confirmed by clinical studies.
